# The characteristic expression pattern of *BMI-1* and *SALL4* genes in placenta tissue and cord blood

**DOI:** 10.1186/scrt199

**Published:** 2013-04-30

**Authors:** Shaohua Chen, Sichu Liu, Ling Xu, Lijian Yang, Zhenyi Jin, Yu Ma, Bo Li, Xiuli Wu, Jianchang Yang, Yupo Ma, Yangqiu Li

**Affiliations:** 1 Key Laboratory for Regenerative Medicine of Ministry of Education, Jinan University, Guangzhou 510632, China; 2 Institute of Hematology, Jinan University, Guangzhou 510632, China; 3 Department of Pathology, BST-9, School of Medicine, The State University of New York at Stony Brook, Stony Brook, NY 11794, USA

**Keywords:** *BMI-1* gene, Cord blood, Placenta, Real-time PCR, *SALL4* gene

## Abstract

**Introduction:**

*SALL4* and *BMI-1* are important factors in hematopoiesis. Placental tissue (PT) and umbilical cord blood (CB) are rich in hematopoietic stem/progenitor cells (HSCs/HPCs), but their *SALL4* and *BMI-1* expression levels remain unknown.

**Methods:**

Real-time PCR was used to determine the expression level of these genes in PT and CB from ten cases, and ten healthy donors were used as controls.

**Results:**

A significantly higher *BMI-1* and *SALL4* gene expression level was found in PT (median: 17.548 and 34.362, respectively) than in cord blood mononuclear cells (CBMCs) (median: 2.071 and 11.300, respectively) (*P* = 0.0001 and *P* = 0.007) and healthy peripheral blood mononuclear cells (PBMCs) (median: 0.259 and 0.384, respectively) (*P* = 0.001 and *P* <0.0001), and their expression level was lower in PBMCs than in CBMCs (*P* = 0.029 and *P* = 0.002). A positive correlation between the *BMI-1* and *SALL4* genes was found in the PT and CB groups, while there was no significant correlation between these genes in the healthy group. There was also no significant correlation between the expression level of each gene in PT and CB.

**Conclusions:**

These results describe the characteristic features of the *BMI-1* and *SALL4* gene expression pattern in placental tissue and cord blood. Placental tissue with higher expression level of both genes may be considered as a potential resource for *SALL4*-related HPC expansion.

## Introduction

Umbilical cord blood (CB) is a valuable alternative hematopoietic stem cells (HSC) source for transplantations for patients who lack a suitable sibling donor [1-3]. It has been demonstrated previously that CB-derived progenitors can express Oct3/4, SRY-related HMG-box 2 (Sox2), Nanog and reduced expression-1 (Rex1), which are pluripotent/multilineage markers and could potentially differentiate into multiple lineages [4,5]. However, different gene expression pattern may determine the use of human CB-derived HSCs/hematopoietic progenitor cells (HPCs) as functional tissues or cells [6].

Sal-like protein 4 (*SALL4*) and B cell-specific MLV integration site 1 (*BMI-1*) are important factors in hematopoiesis and are expressed in hematopoietic stem/progenitor cells (HSCs/HPCs) and myeloid leukemia cells [7]. Placental tissue (PT) and CB comprise rich HSCs/HPCs; however, little is known about the difference in the expression level of *SALL4* and *BMI-1* in PT and CB.

*SALL4*, a newly identified zinc-finger transcription factor that is a member of the *SALL* gene family, was originally cloned based on its sequence homology to *Drosophila spalt* (*sal*) [7,8]. This protein plays important roles in maintaining embryonic stem cells (ESC) pluripotency and HSC/HPC self-renewal properties, and it has been recently proposed for use in CB expansion [9]. Recently, it was demonstrated that *BMI-1* is a direct *SALL4* target gene. *BMI-1* is a member of the polycomb group of proteins, and it was initially identified in *Drosophila* as a repressor of homeotic genes [7,10]. This protein is highly expressed in purified HSCs, its expression declines with differentiation [7,11], and it plays an essential role in regulating adult, self-renewing HSCs/HPCs [7,11-13]. The induction of *SALL4* expression is associated with increased levels of histone H3-K4 and H3-K79 methylation in the *BMI-1* promoter, indicating a novel connection between *SALL4* and the polycomb group proteins [7].

In this study, we sought to characterize the expression pattern of the *BMI-1* and *SALL4* genes in PT and CB.

## Materials and methods

### Samples

Ten placental tissue samples were obtained from full-term deliveries, and umbilical cord blood was obtained at the same time from full-term healthy babies with the mothers’ consent. All human tissue and cell samples were obtained with consent from the human subjects. Peripheral blood samples from ten healthy donors with informed consent were used as control. All of the procedures were conducted according to the guidelines of the Medical Ethics Committees of the Health Bureau of the Guangdong Province of China, and ethical approval was obtained from the Ethics Committee of Medical School of Jinan University for this study.

### Quantitative real-time reverse transcription-polymerase chain reaction (qRT-PCR)

Mononuclear cells were isolated from cord blood samples (CBMCs) and healthy peripheral blood (PBMCs) by Ficoll-Hypaque gradient centrifugation. The placental tissue (approximately 100 mg) was obtained by curettage of the central portion of the placenta (decidua in majority). RNA isolation and cDNA synthesi*s* were performed according to the manufacturer’s protocol [14].

The expression level of *BMI-1*, *SALL4*, and the β2 microglobulin (*β2-MG*) reference gene was determined by SYBR Green I real-time PCR as previously described [15].

### Statistical analyses

Differences in mRNA expression between two groups were analyzed using the Mann-Whitney test. Data are presented as the median. Spearman’s rank correlation analysis was used to analyze the *SALL4* and *BMI-1* mRNA levels in different samples. Differences were considered statistically significant with a *P* <0.05.

## Results and Discussion

**Figure 1:**
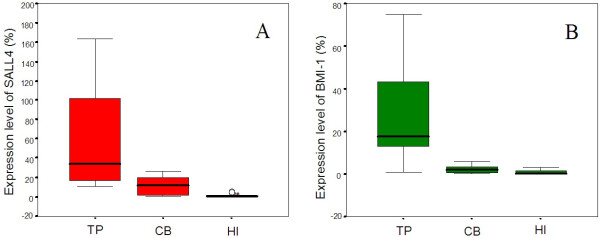
**Expression level of *SALL4 *and *BMI-1. ***Expression level of the *SALL4* (**A**) and *BMI-1* (**B**) genes in placental tissue (PT), cord blood and peripheral blood mononuclear cells (CBMCs and PBMCs, respectively) from healthy individuals (HI). A significant difference was found among the three groups.

Due to its wide availability, low cost and lack of ethical concerns, CB provides an attractive source of stem cells for investigational and therapeutic uses [1-3]. Recently, in the promising field of regenerative medicine, human perinatal stem cells that could be isolated from normally discarded human placentae are of great interest as potential stem cells with clinical applications. Perinatal stem cells are an ideal cell source in terms of availability, the fewer number of ethical concerns, less DNA damage, and so on [16,17]. The biological characteristics of CB-derived HSCs/HPCs were characterized in numerous studies [4,6]. *SALL4* may act as a critical regulator of the fate of hematopoietic cells. In normal bone marrow, *SALL4* is selectively expressed in primitive hematopoietic precursors and is rapidly downregulated following differentiation. Therefore, the *SALL4* gene expression level should be positively correlated with stem cells [7]. However, little is known about the difference in the expression level of *SALL4* in CB; thus, it may be interesting to determine the expression characteristics of this gene in PT collected from the same case as the CB to compare expression pattern differences. In this study, we found that the *SALL4* expression level was higher in PT (median: 34.362) than that in CBMCs (median: 11.300; *P* = 0.007) and PBMCs (median: 0.384; *P* < 0.0001), and a lower *SALL4* expression level was found in PBMCs than CBMCs (Figure 1A; *P* = 0.002). It has been reported that *SALL4*-expanded HSCs/HPCs retain multilineage repopulation and long-term engraftment abilities, which are clinically significant [18]. Thus, *SALL4* could be used to stimulate the large scale *ex vivo* expansion of HSCs/HPCs, and it is particularly interesting whether *SALL4*-rich placental tissue is able to be used as a resource for HSC/HPC expansion.

**Figure 2:**
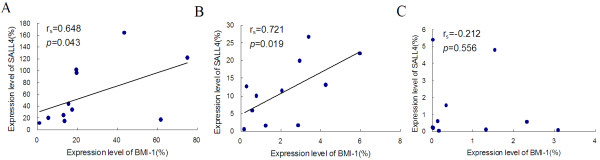
**Correlation of expression level of *SALL-4 *and *BMI-1 *in different samples.** Correlation analysis of the relative expression level of the *BMI-1* and *SALL4* genes in placental tissue (PT) (**A**), cord blood mononuclear cells (CBMCs) (**B**) and peripheral blood mononuclear cells (PBMCs) (**C**).

**Figure 3:**
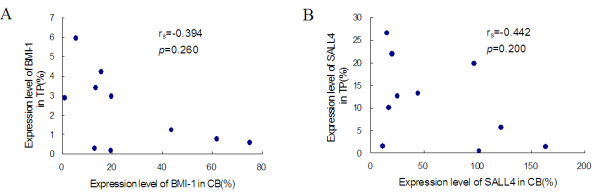
**Correlation of expression level of *BMI-1 *and *SALL4 *between PT and CB.** Correlation analysis of the relative expression level of the *BMI-1* (**A**) and *SALL4* (**B**) genes in placental tissue (PT) and cord blood mononuclear cells (CBMCs).

Because *BMI-1* is a direct *SALL4* target gene [7], *BMI-1* expression may be correlated with that of *SALL4*. Our results also demonstrated that the *BMI-1* gene expression level was significantly higher in PT (median: 17.548) than that in CBMCs (median: 2.071; *P* = 0.0001) and PBMCs (median: 0.259; *P* = 0.001), and its expression level was lower in PBMCs than that in CBMCs (Figure 1B; *P* = 0.029). A positive correlation between the *BMI-1* and *SALL4* genes was found in the PT and CB groups (rs = 0.648, *P* = 0.043 and rs = 0.721, *P* = 0.019, respectively), while there was no significant correlation between these genes in the healthy group (rs = -0.212, *P* = 0.556) (Figure 2). This result further supports the finding of a relationship between the expression pattern of *BMI-1* and *SALL4* in HSC/HPC-rich CB and placental tissue, while PBMCs contain only limited *BMI-1* and *SALL4* expression, which may reflect their expression in different levels of HPCs and myeloid cells [7]. Moreover, this result may indicate that the PT niche contains hematopoietic transcription factors that are able to enhance HPC expansion. It is thought that the expression level of genes in PT and CB derived from the same case may demonstrate a positive correlation; however, interestingly, the expression levels of *BMI-1* and *SALL4* were not significantly correlated between the PT and CB (Figure 3). This result may be related to a difference in the HSC/HPC numbers in CB or may imply that placental tissue-derived HSCs/HPCs have a different *SALL4* and *BMI-1* expression pattern. Further analysis is needed to compare the expression levels of these genes and the percentages of HSCs/HPCs in CB and PT.

## Conclusions

We determined the expression characteristics of *BMI-1* and *SALL4* in placental tissue and cord blood. The results of this study may contribute to a better understanding of the expression characteristics and correlation of *SALL4* and *BMI-1* in placenta tissue. Placental tissue with higher expression level of both genes may be considered as a potential resource for *SALL4*-related HPC expansion.

## Abbreviations

BMI-1: B cell-specific MLV integration site 1 gene; CB: umbilical cord blood; CBMCs: cord blood mononuclear cells; ESC: embryonic stem cells; HPCs: hematopoietic progenitor cells; HSCs: hematopoietic stem cells; PBMCs: healthy peripheral blood mononuclear cells; PT: placental tissue; qRT-PCR: quantitative real-time reverse transcription-polymerase chain reaction; Rex1: reduced expression 1; SALL4: Sal-like protein 4 gene; Sox2: SRY-related HMG-box 2; β2-MG: β2 microglobulin

## Competing interests

The authors declare that they have no competing interests.

## Authors’ contributions

YL, YM and JY contributed to the concept development and study design. SC, SL, LX, ZJ, and YM performed real-time PCR experiments. LY, BL and XW were responsible for the collection of clinical samples. YL, SC, SL and LX coordinated the study and helped in drafting the manuscript. All authors read and approved the final manuscript.
